# Tissue inflammation and NCDs: dietary control, physical exercise and mind body interactions

**DOI:** 10.1186/1753-6561-6-S3-P53

**Published:** 2012-06-01

**Authors:** Fabien De Meester, Agnieszka Wilczynska, Ram B Singh, Douglas W Wilson, Amritpal S Hungin, Daniel Pella, Jan Fedacko, Jarmila Siegelova, Bohumil Fiser, Claudio Galli, Germaine Cornélissen, Franz Halberg

**Affiliations:** 1 Tsim Tsoum Institute, Krakow, 31-007, Poland; 2 School of Medicine and Health, Durham University, Stockton-on-Tees, TS17 6BH, UK; 3 Faculty of Medicine, PJ Safarik University, Kosice, 041 90, Slovakia; 4 Faculty of Medicine, Masaryk University, Brno, 656-91, Czech Republic; 5 Department of Pharmacological Sciences, University of Milan, Milan, 20133, Italy; 6 Chronobiology Center, University Minnesota, Minneapolis, MN 55455, USA

## Background

Cause and effect of dietary constituents on the genesis of NCD epidemics (CVD, diabetes, etc) in the Western developed and some developing countries has been difficult to unravel. It appears that inflammation is a key factor and that in its absence total cholesterol may have an apparently neutral effect on arterial tissues and the myocardium. The Mediterranean-style diet, combined with physical exercise, resembles a notional Palaeolithic diet of a pre-agricultural era in that the ratio of omega-3/omega-6 unsaturated fatty acids is higher than in most Western diets [1]. Furthermore, evidence suggests that this higher ratio can beneficially change brain function and its interaction with gut, liver and heart and influence mind-body interactions. This group, in collaboration with others, seek to test the hypothesis, albeit for a 4y longitudinal psychosomatic randomised placebo control vs. omega-3 intervention trial of 90 subjects, 30 each in adolescent, adult and elderly groups of men and women in Poland, that this higher ratio can not only favourably change the rhythmic characteristics of blood pressure and heart rate, but beneficially improve mental health. However, a 3m feasibility study has been undertaken to test methods and safety associated with the main protocol the underpinning objective being that a return to the original balanced (1:1) ratio of polyunsaturated fatty acids (PUFAs) and/or to a corresponding 25% proportion of ω6 highly unsaturated fatty acids (HUFAs) in plasma/ serum total lipids (ω6:ω3 PUFAs = 1:1 and/or %ω6 in HUFAs = 25) can possibly reduce the risk of developing chronic degenerative diseases towards zero at the population level.

## Materials and methods

Subjects (consented): 5 family-related members + 4 family acquainted subjects were arranged in the three age groups. Clinical (published elsewhere [2]): General medical examination with blood and urine tests; psychological health assessment using Eysenck’s Personality Questionnaire (Polish); body mass composition; ambulatory blood pressure monitoring; blood lipids were the main methods.

## Results

All subjects reported subjective mind and body health improvements concerning cognition, concentration, communication, breathing, general feeling, relief of dizziness and pain confirmed by medical, biochemical and socio-psychological analyses. In all three subgroups, prolonged w-3 HUFA intake appears to maintain the new status, not to further reduce it. The main results were: 1. Daily intake of w-3 HUFA needed to reverse the Omega-6 Status from 75 to 50% (3.35g w-3 HUFA )/day) and then from 50 to 25% (3.35g w-3 HUFA/day) in the blood-tissue of a modern Western population was redefined; 2. Benefits of the presence of a “blunter” (LIPISTASE) of inter-individual genotypic variance were indicated; 3. A reduction in daily intake of omega-6 fatty acids from plant and animal origins is probably needed to augment post-intervention to reach the evolutionary selected blood/tissue ratio; 4. age and gender, body composition, metabolic rate and lifestyle did not substantially affect the linear relationship between the change in w6S and omega-3 intervention dose. Safety appears not to be an issue.

## Conclusions

Pilot data supports, with amendments, the implementation of the 4y longitudinal randomized placebo vs. omega-3 intervention trial.
